# Pro- and anti-inflammatory cytokines Interleukin-6 and Interleukin-10 predict therapy outcome of female patients with posttraumatic stress disorder

**DOI:** 10.1038/s41398-022-02230-3

**Published:** 2022-11-09

**Authors:** Vanessa Renner, Peter Joraschky, Clemens Kirschbaum, Julia Schellong, Katja Petrowski

**Affiliations:** 1 Medical Psychology & Medical Sociology, University Medical Center of the Johannes Gutenberg University Mainz, Mainz, Germany; 2 University Medical Center Carl Gustav Carus, Technische Universität Dresden, Dresden, Germany; 3 Department of Psychology, Institute of Biological Psychology, Technische Universität Dresden, Dresden, Germany; 4 Faculty of Medicine Carl Gustav Carus, Department of General Practice/MK3, Technische Universität Dresden, Dresden, Germany

**Keywords:** Predictive markers, Human behaviour

## Abstract

PTSD patients show alterations of the immune system, mainly a ‘low-grade inflammation’. Psychotherapeutic treatments are meant to reduce symptom burden of PTSD patients but 30–50% of PTSD patients do not benefit from psychotherapy. Therefore, in this study, the predictive effect of cytokine levels on therapy outcome are investigated. Pro- (IL-6) and anti-inflammatory (IL-10) cytokines in female PTSD patients (N = 17) were assessed under acute stress during a Trier social stress test (TSST) before therapeutic treatment. The predictive effects of IL-6 and IL-10 on therapy outcome (SCL_GSI, BDI) after an inpatient psychotherapeutic treatment at the University Medical Center Carl Gustav Carus, Technische Universität Dresden was investigated. Areas under the curve with respect to ground (AUC_G_) and increase (AUC_I_) for IL-6 and IL-10 levels during the TSST were calculated and used as predictors in regression analyses with pre-treatment scores. Models including all three predictors show good model fits (R^2^ = 0.255 to 0.744). Models including AUC_G_ and AUC_I_ scores show superior fits compared with models including pre-treatment scores alone (ΔR^2^ = 0.196 to 0.444). IL-6 AUC_G_ and AUC_I_ scores are significant predictors for post-treatment SCL-GSI and BDI (β = −0.554 to 0.853), whereas IL-10 AUC_G_ significantly predicts SCL-GSI and BDI (β = −0.449 to −0.509). Therefore, pro- and anti-inflammatory IL-6 and IL-10 levels under acute stress before therapy predict therapy outcome of female PTSD patients regarding general symptom burden and depressive symptoms. Future studies should further address the link between inflammation and therapy outcome, especially underlying mechanisms and influencing factors.

## Introduction

Post-traumatic stress disorder (PTSD) develops after being exposed to or having witnessed a life-threatening event. Symptoms of PTSD are re-experiencing the event or parts of it in flashbacks or nightmares, hyperarousal and avoiding aspects related to the traumatic event [1]. Somatically, PTSD patients often show various diseases such as cardiovascular, respiratory, gastrointestinal and autoimmune diseases [2]. Even when controlling for risk factors for somatic diseases such as smoke status or somatic conditions such as hypertension, PTSD patients still show an elevated risk for somatic diseases [2, 3].

Based on these findings, a direct link between psychological disorders and somatic diseases is hypothesized. It is assumed that an alteration of the hypothalamus pituitary adrenal-axis (HPA-axis) and the immune system might promote the development of somatic diseases via cytokines [4]. Cytokines, a family of proteins, mediate immune responses to injury, infection or other organismal stress and are associated with inflammatory diseases, e. g. cardiovascular diseases, cancer, or autoimmune disorders [4, 5]. As described in past studies, an activation of the HPA-axis because of stress triggers a cascade resulting in an immunoregulatory function by inhibiting inflammatory cytokines such as Interleukin (IL-) 6 and increasing production of anti-inflammatory cytokines such as IL-10 [6]. Different studies investigated these mechanisms and found alterations of the HPA-axis and the immune system in PTSD patients showing a blunted HPA-axis stress reactivity compared with healthy controls [7, 8]. Regarding the immune system, mainly a so-called ‘low-grade inflammation’ was found in PTSD patients showing increased inflammatory cytokine levels compared with healthy controls [4]. These elevated levels of IL-6 were found to be associated with worse cognitive functioning of PTSD patients [9]. Therefore, it is supposed that these altered mechanisms of the HPA-axis and the immune system might lead to inflammatory body states in PTSD patients promoting the development of the mentioned diseases.

What remains unclear is, if cytokine levels also influence therapy outcome. 30% of patients with psychological disorders during clinical trials and 65% of patients in routine care do not respond to psychotherapy [10]. 30–50% of PTSD patients do not benefit from trauma-focused therapy sufficiently but show substantial residual symptoms after the end of the therapy [11, 12]. For this reason, past studies investigated aspects influencing therapy outcome and found various predictive factors [13, 14]. Regarding therapy outcome of PTSD patients, neuroimaging data and data of pre-treatment biomarkers among others were used for predicting therapy outcome [12, 15]. Higher bedtime salivary cortisol and lower urinary cortisol before treatment predicted greater symptom reduction [15, 16]. Studies examining the predictive character of cytokine levels on treatment outcome are rather rare. Rhein et al. (2021) assessed IL-6 levels of PTSD patients after a psychosocial stress test before therapy and the severity of depressive, trauma-related, and somatic symptoms 8 weeks after treatment. It was shown that patients with a high reactivity to the stress test, reflected by high IL-6 levels, showed a more negative therapy outcome. This study first showed an association between cytokine levels and therapy outcome [17].

The mentioned studies show that different biomarkers such as cortisol and cytokines influence therapy outcome of PTSD patients [14–16]. Nevertheless, the role of anti-inflammatory cytokines remains unclear and studies addressing the influence of cytokines on therapy outcome are very rare in general. Therefore, in this study, we assess the predictive character of pro-inflammatory IL-6 and anti-inflammatory IL-10 levels after a psychosocial stress test at the beginning of psychotherapeutical treatment regarding therapy outcome in female PTSD patients.

## Methods

### Study sample

Patients were recruited consecutively at the University Medical Center Carl Gustav Carus of the Technische Universität Dresden during the beginning of their inpatient treatment. 17 female PTSD patients between 28 and 58 years old (M = 47.12, SD = 8.52) with fluent German language skills were included in this sample. Exclusion criteria were a lifetime history of substance use disorder, psychotic or bipolar disorder, psychopharmacological or glucocorticoid-containing medication intake (e. g. asthma inhaler, topical cortisone creams), severe medical illnesses (e. g. cancer, autoimmune diseases, diabetes) and pregnancy. The Structured Clinical Interview (SCID-IV) [18] was conducted by trained interviewers for assessing mental disorders based on the DSM-IV-TR. All of the participants did at least show one comorbidity, n = 1 showed the maximum of 5 additional diagnosis to PTSD, most of the patients (n = 9) showed 3 comorbidities. N = 16 PTSD patients did additionally suffer from a major depressive disorder, n = 6 from panic disorder with/ without agoraphobia, n = 9 from social anxiety disorder, n = 8 from somatoform disorder. None of the participants did use contraceptives, n = 14 took medication and n = 10 of them were taking psychotropic drugs. N = 8 patients were married, 3 were divorced, 4 were single and 2 were widowed. 3 of the patients were uncapable for work and 6 of them did smoke. All of the participants provided written informed consent and the study procedure was conducted in accordance with the Declaration of Helsinki and approved by the local Ethics Committee of the Medical Faculty of the Technische Universität Dresden, Germany.

### Psychosocial stress induction and hormone sampling

At the beginning of the inpatient treatment, patients participated in a standardized stress test, the Trier social stress test (TSST). The TSST reliably induces acute moderate psychosocial stress under laboratory conditions [19, 20]. It requires a mock job interview (5 min) and a mental arithmetic task (5 min) of the participants, which are performed in front of a mock selection committee. The TSST took place after 2 pm in the afternoon to account for the circadian rhythm of cytokine secretion. The female participants were tested in the luteal phase of their menstrual cycle. Blood samples were collected via a venous catheter 15 min and 1 min prior to the TSST as well as 1 min, 10 min, 20 min, 30 min, 45 min and 60 min after the TSST and set to coagulate for 30 min at room temperature. Afterwards, the blood samples were centrifuged at 20 °C for 10 minutes at 2500xG RCF. Blood samples were stored at −80 °C before being assayed for cytokines. Serum cytokine concentrations (IL-6 and IL-10) were determined using highly-sensitive ELISA enzyme-linked immunosorbent assays (IBL International GmbH, Germany).

### Therapy procedure

PTSD patients received a multimodal trauma-focused psychotherapy, the mean duration of treatment was 9 weeks (SD = 3.44). Each week, patients participated in two sessions of individual therapy, which included trauma-focused elements such es Eye Movement Desensitization and Reprocessing or trauma-focused cognitive behavioural therapy. Individual therapy sessions were either offered by board-certified psychotherapists or experienced psychologists and were all supervised by an experienced psychotherapist. Furthermore, each week patients received two sessions of group therapy, two sessions of communicative movement therapy, two sessions of art therapy, one session of progressive muscle relaxation, one session of psychoeducation group, one session of training in social competences group and one session of trauma-stabilization group or skills training. If indicated, daily brief sessions with a nurse took place.

### Clinical assessment

Two German language self-report questionnaires were handed out to the study participants for a clinical characterization in the beginning of the therapy and as a measure of therapy outcome at the end of therapy. In both, higher questionnaire scores indicate higher symptom burden. The Symptom Checklist (SCL-90-R) [21, 22] assesses the general psychological symptom burden within the last seven days and consists of 90 items. The global severity index (GSI) reflects the degree of general impairment. The SCL-90-R meets high internal consistencies with Cronbach’s α = 0.97 for the global index. Depressiveness was evaluated with the Beck Depression Inventory II (BDI-II) [23] including 21 items that match the DSM-IV-TR major depression criteria. Internal consistencies (Cronbach’s alpha) are high with α = 0.90 to 0.93.

### Statistical analyses

A power analysis was conducted with StataIC 16 V5 to calculate an appropriate sample size. Therefore, based on a power of 80%, α = 0.05 and R^2^ = 0.50 were assumed. Based on this calculation, a sample size N = 18 was proposed and implemented. One patient had to be excluded because of missing data in the clinical assessment resulting in N = 17. As IL-6 and IL-10 values were skewed, they were subjected to ln-transformations [24]. For IL-10 values, there were in total 5.88% of missing data for different patients and time points. Therefore, missing cytokine values were imputed using predictive means matching over 10 iterations using R with the package *mice* [25]. Pre-/ post-differences in subjective symptom burden (SCL-90_R GSI, BDI) were calculated using paired t-tests. Results were considered significant when p < 0.05. Area under the curve with respect to ground (AUC_G_) and increase (AUC_I_) [26] were calculated for cytokine levels (IL-6, IL-10) during the TSST using StataIC 16 V5. To check for multicollinearity before regression analyses, AUC scores and pre-treatment scores were correlated using Pearson correlations. No correlation exceeded 0.7, so no evidence of multicollinearity was found. At first, in regression analyses pre-treatment scores were used as predictors for therapy outcome. Then, values for AUC_G_ and AUC_I_ were additionally added as predictors for therapy outcome (SCL-90 GSI, BDI). R^2^ greater than | 0.26| indicate a high goodness-of-fit [27]. ΔR^2^ was computed to assess additional benefit from including AUC scores in the regression analyses.

## Results

**Fig. 1:**
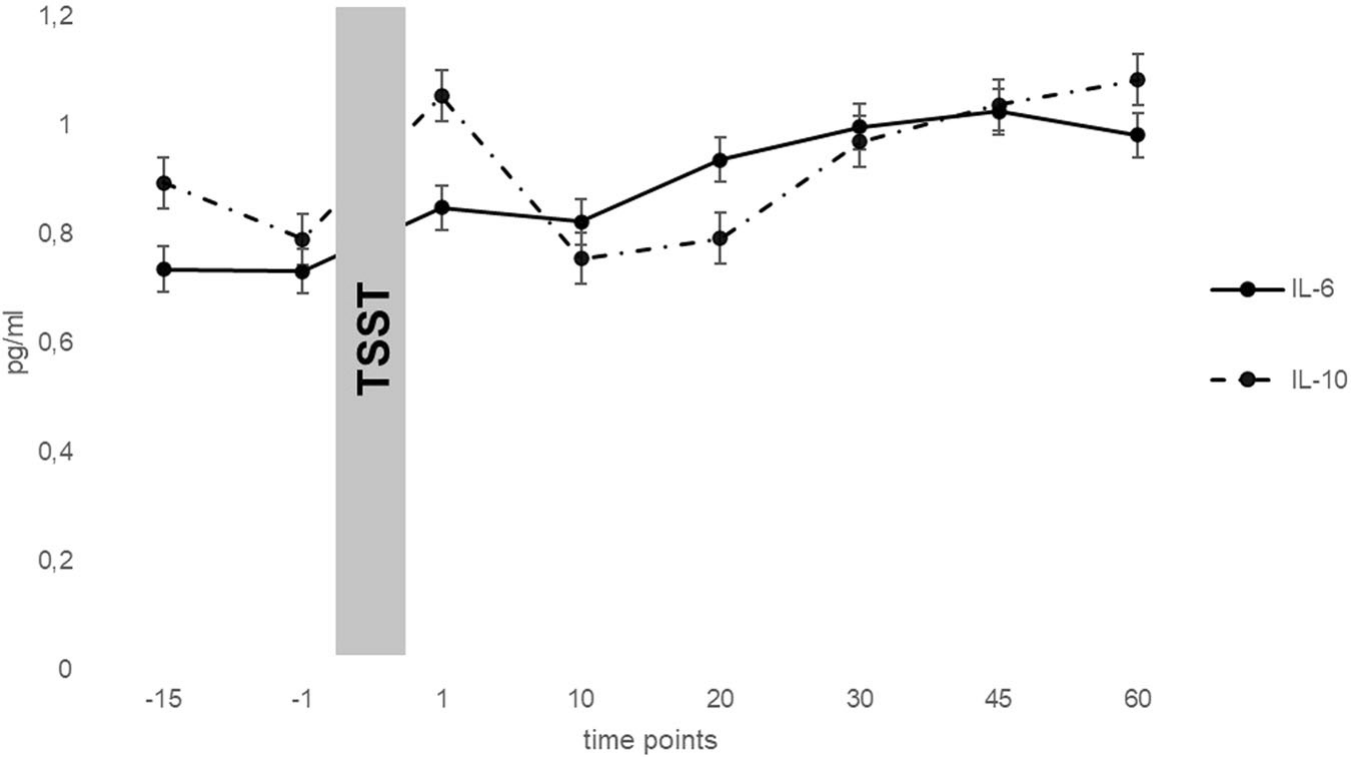
Mean (±SE) IL-6 and IL-10 levels across measurement points of the TSST in PTSD patients. IL-6 Interleukin 6, IL-10 Interleukin 10, TSST Trier social stress test, PTSD posttraumatic stress disorder, time points in minutes.

PTSD patients did show significant lower symptom burden on the BDI after therapy (M = 21.94, SD = 14.87) compared with BDI scores at the beginning (M = 29.81, SD = 11.72; t(15) = 2.50, p = 0.024, d = 1.16). Regarding the SCL-GSI score, there were no significant differences (beginning: M = 1.85, SD = 0.72, end: M = 1.46, SD = 0.97; t(15) = 1.84, p = 0.086). Figure 1 shows cytokine levels (IL-6, IL-10) in PTSD patients for different time points before and after the TSST, Table 1 includes means and standard deviations of IL-6 and IL-10 levels for each time point before and after the TSST. Further analyses of these patterns in comparison with depressive patients and healthy controls can be derived from a former article [28]. Results of Pearson correlation of AUC scores and pre-treatment scores can be derived from Table 2.

**Table 1:** Means and standard deviations of logarithmized IL-6 and IL-10 levels for all time points during the TSST for PTSD patients.

	IL-6 *Mean (SD)*	IL-10 *Mean (SD)*
−15 min	0.76 (0.31)	0.86 (0.55)
−1 min	0.76 (0.27)	0.76 (0.50)
+1 min	0.85 (0.34)	1.05 (0.52)
+10 min	0.82 (0.42)	0.75 (0.45)
+20 min	0.94 (0.41)	0.79 (0.48)
+30 min	0.99 (0.43)	0.97 (0.46)
+45 min	1.02 (0.44)	1.04 (0.44)
+60 min	0.98 (0.41)	1.08 (0.44)

*Notes*. *SD* standard deviations, *min* minutes.

**Table 2:** Pearson correlation of AUC scores and pre-treatment questionnaires.

	AUC_G_ IL-6	AUC_I_ IL-6	AUC_G_ IL-10	AUC_I_ IL-10	SCL-GSI_b	BDI_b
AUC_G_ IL-6	1	0.512*	−0.640**	0.082	0.067	0.116
AUC_I_ IL-6	0.512*	1	−0.510*	0.220	−0.439	−0.392
AUC_G_ IL-10	−0.640**	−0.510*	1	−0.222	0.182	−0.026
AUC_I_ IL-10	0.082	0.220	−0.222	1	−0.408	0.019
SCL-GSI_b	0.067	−0.439	0.182	−0.408	1	0.497*
BDI_b	0.116	−0.392	−0.026	0.019	0.497*	1

*Note*. *AUC*_*G*_ Area under the curve with respect to ground, *AUC*_*I*_ Area under the curve with respect to increase, *SCL_GSI* Global severity index of the Symptom Checklist, *BDI* Beck Depression Inventory, *b* pre-treatment scores (beginning of therapy), *r* Pearson correlation coefficient.

*p < 0.05.

**p < 0.01.

Bold values denote significant *p* values.

In regression analyses, when predicting therapy outcome by pre-treatment scores, SCL-GSI pre-treatment scores significantly predict SCL post-treatment scores (F(1,15) = 5.13, p = 0.039, R^2^ = 0.255) and also BDI pre-treatment scores significantly predict BDI scores after treatment (F(1,15) = 8.05, p = 0.013, R^2^ = 0.349). Results from regression analyses including pre-treatment scores and AUC scores as predictors for therapy outcome can be derived from Table 3. Model fits are consistently higher than R^2^ = 0.26, reflecting good model fits. 69.9% of the variance of SCL-GSI post-treatment scores is explained by IL-6 AUC scores and pre-treatment SCL-GSI scores. In this model, AUC_G_ (β = 0.853, p = 0.001) and AUC_I_ (β = −0.554, p = 0.024) are significant predictors for SCL-GSI post-treatment scores whereas SCL-GSI pre-treatment scores are not (β = 0.204, p = 0.287). 74.4% of the variance of BDI post-treatment scores is explained by IL-6 AUC scores and pre-treatment BDI scores. Also in this model, BDI pre-treatment scores do not significantly predict therapy outcome (β = 0.302, p = 0.104) whereas AUC_G_ (β = 0.814, p = 0.001) and AUC_I_ (β = −0.449, p = 0.044) coefficients show significant predictions. For IL-10, explained variances are smaller but the models still show a good model fit (SCL-GSI: R^2^ = 0.571, BDI: R^2^ = 0.545). Regarding SCL-GSI, AUC_G_-IL10 (β = −0.509, p = 0.018) and SCL-GSI pre-treatment scores (β = 0.686, p = 0.005) show significant regression coefficients, whereas AUC_I_-IL10 does not significantly predict SCL-GSI post-treatment scores (β = 0.686, p = 0.319). The same pattern was found in the regression model predicting BDI post-treatment scores (AUC_G_-IL10 β = −0.449, p = 0.036; AUC_I_-IL10 β = −0.158, p = 0.425; BDI pre-treatment scores β = 0.572, p = 0.009). ΔR^2^ were between 0.196 for predicting BDI post-treatment scores with IL-10 AUC scores and pre-treatment scores as predictors and 0.444 for predicting SCL_GSI post-treatment scores with IL-6 AUC scores and pre-treatment scores.

**Table 3:** Predictors of SCL_GSI and BDI scores after therapy in regression analyses.

		β	SE	F	p	R^2^
SCL_GSI_e						
Model 1	SCL_GSI_b	0.505*	0.309	5.13	0.039	0.255
Model 2	IL-6: AUC_G_ IL-6: AUC_I_ SCL_GSI_b	0.853** −0.554* 0.204	0.007 0.015 0.256	10.07	0.001	0.699
Model 3	IL-10: AUC_G_ IL-10: AUC_I_ SCL_GSI_b	−0.509* 0.209 0.686**	0.007 0.007 0.278	5.76	0.010	0.571
ΔR^2^	Model 2 – Model 1 Model 3 – Model 1		0.444 0.316			
BDI_e						
Model 1	BDI_b	0.591*	0.275	8.05	0.013	0.349
Model 2	IL-6: AUC_G_ IL-6: AUC_I_ BDI_b	0.814** −0.449* 0.302	0.102 0.219 0.228	12.57	<0.001	0.744
Model 3	IL-10: AUC_G_ IL-10: AUC_I_ BDI_b	−0.449* −0.158 0.572**	0.110 0.107 0.247	5.19	0.014	0.545
ΔR^2^	Model 2 – Model 1 Model 3 – Model 1		0.395 0.196			

*Note*. *AUC*_*G*_ Area under the curve with respect to ground, *AUC*_*I*_ Area under the curve with respect to increase, *SCL_GSI* Global severity index of the Symptom Checklist, *BDI* Beck Depression Inventory, *b* pre-treatment scores (beginning of therapy), *e* post-treatment scores (end of therapy).

*p < 0.05.

**p < 0.01.

## Discussion

In this study, the predictive value of IL-6 and IL-10 cytokine levels with regard on therapy outcome was assessed. It was found that IL-6 and IL-10 levels under acute stress in the beginning of the therapy predict therapy outcome regarding general symptom burden and depressive symptoms. Higher levels of IL-6 under acute stress before therapy are associated with higher general symptom burden and more severe depressive symptoms after therapy, whereas higher levels of IL-10 under acute stress before therapy are associated with reduced general symptom burden and less depressive symptoms after therapy. A high reactivity of IL-6 during acute stress before therapy predicts reduced general symptom burden and less depressive symptoms after therapy. The finding of predictive effects of IL-6 regarding therapy outcome is in line with the rarely existing literature [17] and adds further evidence to the predictive role of IL-6 and new insights in the predictive role of IL-10 regarding therapy outcome in female PTSD patients.

These results seem to emphasize the pro-inflammatory role of IL-6 suggested to promote inflammatory body states and the anti-inflammatory role of IL-10 [6]. Whereas higher levels of IL-6 seem to predict worse therapy outcome in this study, higher levels of IL-10 before therapy predict better therapy outcome. Additionally, the reactivity to an acute stressor plays an important role as a high IL-6 reactivity under acute stress predicts an advantageous therapy outcome. Therefore, as past studies show, the immune system of PTSD patients shows alterations compared with healthy controls and this altered immune system also seems to influence therapy outcome as results in this study suggest. One possible link might be the influence of elevated IL-6 levels on cognitive functioning of PTSD patients [29]. PTSD patients showed significantly higher IL-6 levels compared with healthy controls and significantly worse performance in memory, language and attention tasks [29]. As these functions also play an important role during psychotherapeutic treatment, an impairment could influence the therapy process and outcome, which is why this link should be investigated in future studies. As the role of IL-10 was rarely examined in past studies, this study again emphasizes the importance to also take anti-inflammatory cytokines such as IL-10 into account when assessing the role of the immune system in PTSD patients.

Based on these findings, the question arises how the immune system can be influenced and if this ‘low-grade inflammation’ of PTSD patients can be reduced or normalized. In a meta-analysis it was found that especially cognitive behavioral therapy (CBT) lowers levels of pro-inflammatory cytokines, increase immune cells and increase natural killer cell activity. Moreover, CBT was still associated with enhanced immunity six months after treatment [30]. Therefore, CBT seems to have a positive long-term effect on inflammatory states. If this influence is also present in PTSD patients, needs to be addressed in future studies. Another possibility of influencing elevated cytokine levels of PTSD patients is psychopharmacological treatment. Here, several new approaches targeting cytokine levels as alternatives to the known serotonin-reuptake-inhibitor treatments are discussed. For example, glucocorticoids could inhibit the expression of cytokines such as IL-6 [31, 32]. Also, endocannabinoid signaling was shown to have anti-inflammatory effects, which is why an elevation of endocannabinoid signaling with synthetic cannabinoids is discussed to be effective. Studies already found positive effects of endocannabinoids on trauma-associated nightmares and general PTSD symptoms [33, 34].

This study shows predictive effects of elevated IL-6 and IL-10 levels under acute stress on therapy outcome of PTSD patients. Nevertheless, there are some limitations. The study is slightly underpowered as one patient needed to be excluded because of missing data. Therefore, interpretation of the results needs to be made with caution. In this study, only female participants were assessed. On one hand, the prevalence of PTSD is higher among women compared with men and more women seek for trauma-related treatment [35]. On the other hand, it is known that there are gender differences in the immune response of individuals under acute stress [36]. As the sample size is quite small, we tried to control for as many influencing factors as possible (e. g. differences in immune response between genders, menstruation) in advance to not include too many control variables in the calculations and therefore reduce power of our study. 16 out of 17 patients suffered from a major depression additionally to PTSD. In general, PTSD patients often suffer from other mental disorders [37] which makes it difficult to include patients only suffering from PTSD and would affect the generalizability of the results. The general symptom burden assessed via the SCL-GSI did not change significantly through therapy. On one hand, the 9-week treatment is quite short for significantly reducing symptom burden of patients with multiple diagnosis. On the other hand, patients received a trauma-focused therapy addressing the traumatic symptoms and making the patients more aware of their traumatic experiences. This often leads to a higher symptom burden at first, which does not necessarily decrease through therapy to a smaller symptom burden than before treatment. Additionally, in this study one representative for pro- and anti-inflammatory cytokines was assessed, which gives a first insight of predictive effects of these cytokines regarding therapy outcome but for more general conclusions and further insights, additional parameters of the immune system should be assessed.

To our knowledge this is the first study investigating predictive effects of pro- and anti-inflammatory cytokines on therapy outcome in female PTSD patients. The present study showed that pro-inflammatory IL-6 and anti-inflammatory IL-10 levels under acute stress before therapy predict therapy outcome of female PTSD patients regarding general symptom burden and depressive symptoms. These findings emphasize the importance of the often-shown inflammatory state of PTSD patients and its influence on therapy outcome. Future studies should address underlying mechanisms of the link between inflammation and therapy outcome and find ways to ensure a greater benefit from psychotherapy for PTSD patients with low-grade inflammations.
